# Prevalence of Antenatal Depression and Associated Risk Factors among Pregnant Women Attending Antenatal Clinics in Abeokuta North Local Government Area, Nigeria

**DOI:** 10.1155/2016/4518979

**Published:** 2016-08-18

**Authors:** Okechukwu Thompson, IkeOluwapo Ajayi

**Affiliations:** Department of Epidemiology and Medical Statistics, Faculty of Public Health, College of Medicine, University of Ibadan, Ibadan 200212, Nigeria

## Abstract

*Objective*. The prevalence of antenatal depression (AD) and associated risk factors among pregnant women attending antenatal clinics in Abeokuta North Local Government Area, Nigeria, was determined.* Methods*. A descriptive cross-sectional survey was conducted, interviewing 314 pregnant women selected by multistage sampling technique from among those attending antenatal clinics. Information was collected using structured questionnaire and a screening tool, Edinburgh Postnatal Depression Scale (EPDS), to assess probable depression.* Results*. The prevalence of antenatal depression was 24.5%. There were significant associations between antenatal depression and attending public health facility (*P* = 0.000), young maternal age (*P* = 0.012), single marital status (*P* = 0.010), not having formal education (*P* = 0.022), large family size (*P* = 0.029), planned pregnancy (*P* = 0.014), coexisting medical conditions (*P* = 0.034), history of previous caesarian section (*P* = 0.032), drinking alcohol during pregnancy (*P* = 0.004), and gender based abuse (*P* = 0.001). On health seeking behaviour for antenatal depression among depressed pregnant women, most, 68.9%, consulted their husbands about their symptoms; 57.3% took the decision to get treatment from doctors, and 52% sought prayer in the church.* Conclusion*. Antenatal depression is prevalent in this study population. Interventions to address its risk factors should be carried out and physicians should suspect depression in pregnant women reporting alcohol use and gender abuse.

## 1. Introduction

Depression is a mood disorder which is characterized by prolonged sadness and marked loss of interest in daily activities as core symptoms lasting for one week or more. Other symptoms are numbness, feeling inadequate and worthless, feeling irritable and resentful, insomnia, appetite changes, decreased energy, lack of concentration and poor memory, and thoughts of committing suicide or abortion [1]. Depression is a common mental illness which is ranked the third most prevalent moderate and severe disabling condition globally by the World Health Organization [2]. When it occurs in pregnancy (antenatal depression) it can be a forerunner to postpartum depression.

Women have a lifetime risk of about 1 in 8, and it is most prevalent during their reproductive years [3]. The aetiology of depression is unknown, but it is thought that neurobiological and environmental factors combined with genetic predisposition are influential [4]. Risk factors include history of mood or anxiety disorders, history of postnatal depression, history of postmenstrual dysphoric disorder, family history of perinatal psychiatric illness, history of childhood abuse, low income, poor social support, unplanned pregnancy, single motherhood, large number of existing children, domestic violence or relationship conflicts, and young age [5].

Pregnancy and depression affect each other. Pregnancy is a major psychological, as well as, physiological event. With an excess of chronic life stressors, women may find themselves unable to cope with the additional demands of pregnancy. Many women, particularly those living in poverty or already with dependent children, may view pregnancy with negative feelings. Issues or memories surrounding poor parenting or abuse women have suffered may reassert themselves and cause distress. Relationships are often under pressure because domestic violence increases during pregnancy [1].

Pregnancy-related sex steroids increase activation of hypothalamic-pituitary-adrenal (HPA) axis which is associated with depression [1]. This explains the physiologic aspect of depression in pregnancy. During pregnancy, extensive hormonal changes occur [6]. Corticotrophin Releasing Hormone (CRH) and estradiol regulate HPA axis, causing increased secretion of glucocorticoid cortisol by the adrenal cortex. Elevated cortisol levels inhibit estradiol synthesis and actions. So, pathogenic low levels of plasma cortisol have been associated with melancholic depression, eating disorder, chronic alcoholism, and suicidal tendencies. Target at reduction of HPA activity leads to remission of depression [7].

The prevalence of prenatal depression is estimated to be 10–15% in developed countries and 19–25% in economically poorer countries [1]. In Nigeria, there is paucity of work done on prenatal depression, but of the few works done one published work shows prevalence of antenatal depression (for third trimester only) to be 8.3% [8].

Depression is one of the most prevalent psychiatric conditions in the community. However, it is neither well recognized nor adequately treated in clinical practice [9]. Multiple reasons exist for this inadequate treatment. Patients fail to recognize the symptoms, underestimate the severity, and are reluctant to seek care. They may be noncompliant with their medications, and financial challenges may prohibit the seeking of medical assistance. Health care providers may have inadequate awareness or education about depression and limited training in that area [10, 11].

Antenatal depression has been implicated in nutritional deprivation and poor maternal weight gain during pregnancy. These are associated with intrauterine growth retardation (IUGR) and low neonatal birthweight [12]. Intrauterine growth retardation (IUGR) is a major cause of perinatal mortality and morbidity and an important cause of developmental impairment in later life.

A study has found that there is an association between antenatal depression and labour complications such as prolonged labour, peripartum complications, postpartum complications, and nonvaginal delivery [13].

In addition, some depressed pregnant women engage in smoking of cigarettes and drinking alcohol. This dangerous habit affects the development of the fetus and can cause miscarriages, intrauterine deaths, and intrauterine growth restrictions [14]. Some may manifest suicidal tendencies [15]. Rates of suicidal ideation among depressed obstetric patients have ranged from 3% in Finland [15] to 17.6% in USA [16].

High prevalence of antenatal depression may affect the millennium development goals which are decreasing child mortality (MDG 4) and improving maternal health (MDG 5), hence the need to explore the prevalence and risk factors of antenatal depression in order to provide evidence on the burden and for planning intervention.

Although researches have been carried out on antenatal depression worldwide, there is paucity of work done on it in Nigeria and Abeokuta in particular. A research which was carried out in Nigeria [8] focused on antenatal depression among pregnant women in their third trimester. However, this study focused on antenatal depression in all trimesters among pregnant women.

Another important reason for conducting this study was to contribute to the body of knowledge of antenatal depression and identify the most prevalent predictors of antenatal depression among pregnant women since this condition is neither well recognized, thoroughly studied, nor properly treated clinically [9].

Therefore, this study aimed to determine the prevalence of antenatal depression and associated risk factors among pregnant women in Abeokuta North Local Government Area (LGA), Ogun State. In addition, health seeking behaviour for antenatal depression among the women was explored.

## 2. Materials and Methods

### 2.1. Study Area/Setting

Abeokuta North LGA is a local Government Area in Ogun State of Nigeria. It has a population of 201,329 and the population of child bearing women (15–49 years) is 51,203 at the 2006 census [17]. Abeokuta LGA has 15 wards.

The people are predominantly farmers, most of whom engage in cultivation of arable crop, while some engage in livestock and fishing. In recent times, the people of the area are involved in quarry business, artisan works, and handicrafts such as tie and dye making and pottery [18]. There are about 22 functional health facilities (both private and public) in the LGA. There are two tertiary health institutions (Federal Neuropsychiatric Hospital, Aro, and Olabisi Onabanjo Teaching Hospital, Saje Annex) and six primary health centers in the LGA. There are also many private hospitals and maternity homes. The antenatal clinics in the health facilities are run by doctors and nurses. There are consultant Obstetricians and Gynaecologists, resident doctors, and medical officers in some of these hospitals especially the teaching hospitals. Each primary health center is run by nurses and a doctor but there are few centers without a doctor. The private maternity homes are run by nurses, traditional birth attendants, and community health extension workers (CHEW). Each health facility has fixed days for their ANC service. Doctors and nurses run antenatal clinics where comprehensive obstetric care is offered such as caesarian sections, ultrasonography, and blood transfusion service. Health education is also offered.

### 2.2. Study Design

This was a cross-sectional study.

### 2.3. Study Population

This included all pregnant women attending Antenatal Clinics in Abeokuta North LGA. Any client registering for ANC service on the day of data collection at any facility was included. Pregnant women with physical disabilities such as deafness and dumbness as well as those with a history of or ongoing mental illness/retardation were excluded.

### 2.4. Sample Size Determination

The study sample size of 276 was calculated using sample size formula for cross-sectional study with a prevalence of 8.7% from a study in Nigeria by Adewuya et al., 2007, precision of 5%, and standard normal deviate of 1.96 at 95% confidence intervals. A nonresponse rate of 15% and design effect of two were considered.

### 2.5. Ethical Considerations

This study was approved by the Ogun State Ministry of Health Ethical Board in October 2015. Participants had the right to accept or decline request to participate. Written informed consent was obtained from the participants and their confidentiality was preserved. They were assured of getting the benefits of valuable information about their health, appropriate health advice on how to manage their conditions, and final feedback at the end of the project.

### 2.6. Sampling Technique

Multistage cluster sampling technique was used.

In Abeokuta North Local Government Area, there were about 22 health facilities that offered antenatal care (ANC) services.

*1st Stage*. These health facilities were stratified into three according to their levels of care. Each level was substratified into public and private health facilities. So, based on the level of care, the health facilities were grouped as follows:

1. Primary health facilities: they were divided into public and private. With regard to public health facilities, there were six community health centers, while, for the private, there were nine private maternity clinics.
2. Secondary health facilities: there were no secondary public health facilities (general or state hospitals) in Abeokuta North LGA. However, there were six private hospitals that offered ANC services.
3. Tertiary health facilities: there are two tertiary health institutions in Abeokuta North LGA, namely, Neuropsychiatric Hospital, Aro, and Olabisi Onabanjo University Teaching Hospital (OOUTH), Saje Annex. Of these two, only OOUTH Saje Annex offered ANC services.

Using equal allocation random sampling, the sample size of 314 was shared among the three tiers of health facilities, giving 104 for each tier.

*2nd Stage*. Selection of health facilities from stratified groups was done through simple random sampling technique.

At the primary level, using proportionate allocation ratio of 2 : 3 for primary health centers to maternity clinics, two primary health centers were randomly selected from the six health centers and three maternity clinics were selected randomly from the nine private maternity clinics.

At the secondary level, only private hospitals were sampled since there was no functional public (government) hospital. Two out of the six private hospitals offering ANC services were randomly selected.

At the tertiary level, only OOUTH Saje Annex offered ANC services. So, it was selected.

*3rd Stage*. At the primary level, the allocated sample size was 104. This was equally divided between public (primary/community health centers) and private centers (maternity clinics). So, 52 willing participants were interviewed each from primary health centers and private maternity clinics. The allocated sample size of 52 was divided between the selected primary health centers. So the sample size for each primary health center was 26. The private maternity clinics were three in number; hence the allocated sample size of 52 was divided into three. So, the sample size for each was approximately 18.

At the secondary level, there were two private hospitals. The sample size for each was 52.

At the tertiary level, 104 willing participants were surveyed from the only institution offering antenatal care.

*4th Stage*. In each health facility, during the day of antenatal clinic, the total number of the pregnant women in attendance was determined. If the total number of women in attendance was equal to the allocated sample size, all were selected for interview after obtaining their consent. If the number was more than the allocated sample size, then simple random sampling technique (balloting) was used to select the required number of participants. If the number in attendance was less than the allocated sample size, all were selected, and repeat visit was made on another antenatal clinic day to complete selection. There were no refusals due to proper explanation of the project to the participants before distribution of the questionnaires.

### 2.7. Data Collection Instrument

A questionnaire was developed by the investigator borrowing questions from past studies instruments and knowledge of antenatal depression. In addition, the Edinburgh Postnatal Depression Scale was adopted.

The questionnaire had three sections. The first section focused on sociodemographic characteristics such as age, marital status, ethnicity, occupation, level of education, family size, and social as well as obstetrics history. The second section was on risk factors of depression. In addition the Edinburgh Postnatal Depression Scale (EPDS), a screening instrument to detect depressive symptoms, is used by Epidemiologists and researchers as a substitute for clinical diagnosis of Major Depressive Disorder (MDD). The EPDS is a ten-item self-report scale which was designed in 1987 and was originally meant for postnatal depression. It has since been validated for use in both pregnant and nonpregnant women. The maximum value for EPDS is 30 while the minimum is 0 [19, 20]. EPDS score of >11 is suggestive of clinical depression. The third section of the questionnaire centered on health seeking behaviour for antenatal depression. The questionnaire was developed in English and translated to Yoruba and also translated back to English to ensure adequate translation. A pretest of the questionnaire was carried out in a randomly selected health facility in Abeokuta South LGA. The questionnaire was pretested among 16 pregnant women attending ANC at a health facility in another LGA in Ogun state. It was an interview-administered questionnaire. Both English and Yoruba versions were used depending on the preference of the participants. Six trained research assistants conducted the interview.

### 2.8. Data Analysis

Data collected were entered and stored in a password-protected computer. Statistical Package for Social Science SPSS version 16.0 was used for data entry and analysis. Data cleaning was done prior to analysis. Descriptive statistics such as percentages, means, and standard deviation and range were used to summarize the data. Student's *t*-test and Chi square were used to determine association between means of continuous variables and categorical variables, respectively. Linear multiple regression analysis was used to determine the predictors of antenatal depression from a set of factors significant at bivariate analysis (*P* < 0.05). The dependent variable was antenatal depression and predictors and independent variables were the sociodemographic and obstetric variables such as marital status, age, single parenthood, and unplanned pregnancy. Test of association was carried out at 5% level of significance.

The wealth scores were calculated from respondents' household possession using principal component analysis and grouped into five (quintiles) to give the wealth index. The rich and the very rich had positive wealth score, the average had zero wealth score, and the poor and the very poor had negative wealth score.

With regard to social support, five questions were asked about people in each respondent's life who provided her with help or support. Then, using Sarason et al.'s [21] method of calculating social support questionnaire number score (SSQN), the SSQ number scores for all participants were determined and grouped into three. SSQN score < 1 stood for low social support, SSQN score = 1 stood for average social support, and SSQN score > 1 stood for high social support.

## 3. Results

### 3.1. Sociodemographic Characteristics of Respondents

Table 1 shows the frequency distribution of the sociodemographic characteristics of study respondents. The mean age of the women was 27.3 ± 5.3 years. Majority, 308 (98.1%), of them were Yoruba, and 18 (5.7%) and 292 (93.0%) were single and married, respectively. Those with no formal education were seven (2.2%), while those who had primary education only were 75 (24.0%); those with secondary education were 158 (50.6%) and those who attained tertiary level of education were 72 (23.1%). Two hundred and thirty (78.5%) out of 293 who responded when asked about their type of family were from monogamous families while 61 (20.8) were from polygamous families. Respondents who had small family size (1–4 persons) were 204 (68.9%), average family size (5 persons) were 41 (13.9%), and large family size (≥6 persons) were 51 (17.2%). According to occupational status, the least were the civil servants who totaled six (1.9%), while traders had the highest proportion, 174 (55.9%). According to the wealth index of the study participants, the very rich constitute the lowest proportion, 10 (3.2%), while the poor had the highest proportion, 131 (41.7%). Table 1 also shows social experience, behaviour, and lifestyle of the study participants. One (0.3%) out of 298 respondents smoked and 17 (5.8%) out of 294 respondents affirmed they drank alcohol. According to the type of alcohol taken by these 17 respondents, five (29.4%) took beer, six (35.3) took wine, and six (35.3%) took spirit/liquor. Two (11.8%) took alcohol for medicinal reasons, while 15 (88.2%) took it for pleasure. According to social support, those with low social support (SSQN < 1), average social support (SSQN = 1), and high social support (SSQN > 1) were 29 (9.2%), 216 (68.8%), and 69 (22.0%), respectively. When asked about any forms of abuse they had received from their husbands/lovers or any male gender, six (1.9%) had been raped, 18 (5.7%) had been physically assaulted, 15 (5.1%) had been threatened/intimidated, and 16 (5.1%) had been deprived of financial support. Considering their childhood experience while growing up, 239 (76.1%) mentioned they had pleasant experience, 17 (5.4%) had unpleasant experience, and the rest, 58 (18.5%), indicated that they do not know.

### 3.2. Obstetric History of Respondents

Table 2 shows results on history of index pregnancy and past gynaecological and obstetric history of the respondents. The age of pregnancy of each respondent was calculated from her last menstrual period (LMP) and expected date of delivery (EDD), and this was confirmed with her obstetric ultrasound scan result. The mean gestational age of pregnancy at time of interview was 6.3 ± 2.2 months. Respondents in their first (1–3 months), second (4–6 months), and third (7–9 months) trimesters were 51 (16.3%), 92 (29.4%), and 170 (54.3%), respectively. When asked if the index pregnancy was a planned one, 236 (75.4%) affirmed it was expected and planned for, while 77 (24.6%) did not plan for it. With regard to coexisting medical conditions, 293 (96.1%) out of 305 did not have any medical condition in pregnancy, while those with varied medical conditions were 12 (3.9%). According to the number of times each participant had become pregnant (gravidity), 71 (23.0%) and 238 (77.0%) were primigravida and multigravida, respectively. Considering the parity (the number of childbirths above 28 weeks irrespective of outcomes), nulliparous, primiparous, and multiparous respondents were 91 (29.6%), 93 (30.3%), and 123 (40.1%), respectively. Eighty-five (28.1%) out of 303 respondents had procured abortions or had miscarriages in the past. According to the history of previous childbirths, a total of 172 (47.9%) were male babies, while 187 (52.1%) were females. According to mode of deliveries, 349 (97.2%) babies were delivered via vaginal route, while 10 (2.8%) were born through caesarian section. Considering the weight of babies at birth as provided by the respondents, babies with normal birth weight were 351 (97.8%), while those with low birth weight and high birth weight were 6 (1.7%) and 2 (0.6%), respectively. All babies were said to have been breastfed.

### 3.3. Edinburgh Postnatal Depression Scale Screening Test Results

The results of screening carried out among participants using Edinburgh Postnatal Depression Scale (EPDS) showed that seventy-seven (24.5%) scored 12 and above which was suggestive of depression while the rest, 237 (75.5%), scored below 12. The overall prevalence of antenatal depression was 24.5%. Prevalence of antenatal depression in first, second, and third trimesters was 27.5%, 25%, and 23.5%, respectively.

### 3.4. Associations between Respondent's Characteristics and Antenatal Depression Based on EPDS Scores

#### 3.4.1. Associations between Sociodemographic Variable and Antenatal Depression

Table 3 shows associations between sociodemographic variables and antenatal depression. Prevalence of antenatal depression was highest among those women attending antenatal clinic in a health facility offering tertiary level of care, followed by those receiving antenatal care at health facilities offering primary level of care. When level of care was further analyzed in relation to depression by stratifying heath facilities into private and public subgroups, prevalence of antenatal depression was lower in all private health facilities. There were significant associations between attending public health (*P* < 0.0001), maternal age of 15 to 20 years (*P* = 0.012), being single (*P* = 0.010), having formal education (*P* = 0.022), and large family size (*P* = 0.029) which were significantly associated with antenatal depression. There were significant associations between drinking alcohol during pregnancy and antenatal depression (*P* = 0.004) and gender based abuse and antenatal depression (*P* = 0.001). There was no significant association between social support and antenatal depression (*P* = 0.329), but prevalence of antenatal depression was highest among those with low social support. There was no association between childhood experience and antenatal depression (*P* = 0.122), though prevalence was higher among those with unpleasant childhood experience.

#### 3.4.2. Associations between Obstetric/Gynaecological Variables and Antenatal Depression

Table 4 shows associations between obstetric variables and antenatal depression. Unplanned pregnancy (*P* = 0.014), having coexisting medical condition (*P* = 0.034), and past history of caesarean section (*P* = 0.032) were significantly associated with antenatal depression. There was no association between age of pregnancy and antenatal depression (*P* = 0.845), but prevalence of antenatal depression decreased slightly with increasing trimester. There were no associations between gravidity and antenatal depression (*P* = 0.809), parity and antenatal depression (*P* = 0.344), and abortions/miscarriages and antenatal depression (*P* = 0.546).

### 3.5. Predictors of Antenatal Depression

When the factors found significantly associated with antenatal depression were put in logistic regression model, gender based violence (AOR = 4.3, 95% CI: 2.1–8.9), attending public health facilities (AOR = 5.0, 95% CI: 2.5–9.9), and drinking alcohol in pregnancy (AOR = 5.1, 95% CI: 1.7–14.9) were predictors of antenatal depression (Table 5).

### 3.6. Health Seeking Behaviour for Antenatal Depression among Depressed Respondents

**Figure 1:**
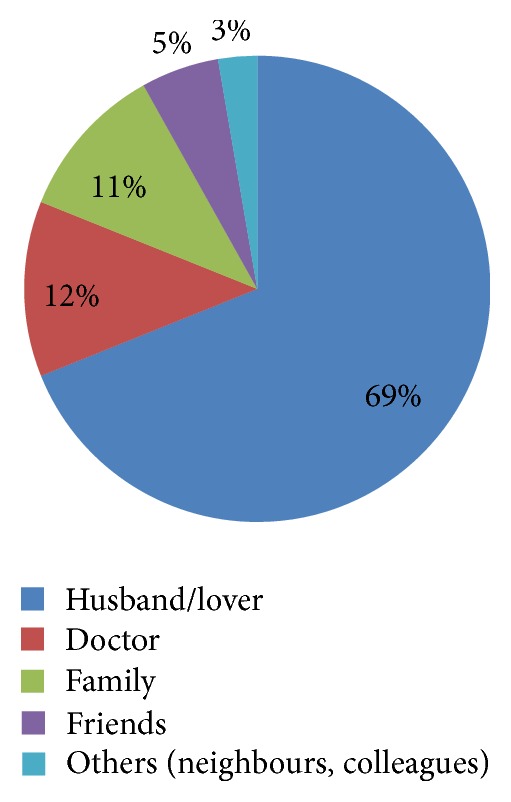
People respondents consulted when they were sad or lost interest in their routine activities (*N* = 74).

**Figure 2:**
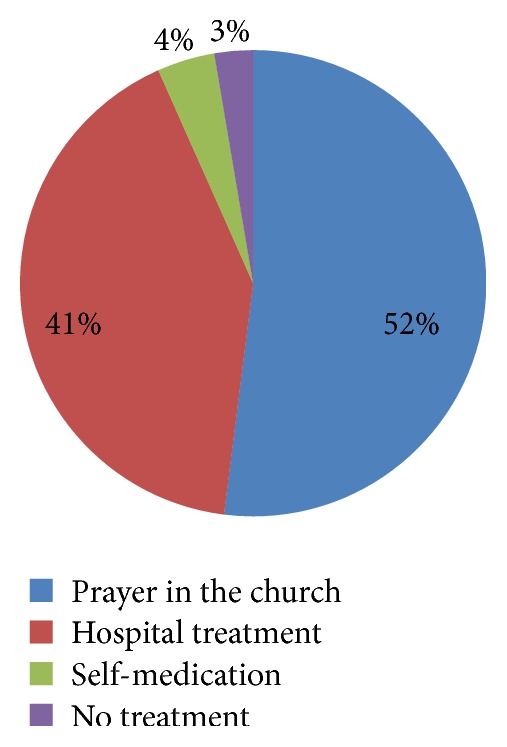
Forms of treatment respondents sought when they were sad or lost interest in routine activities for over one week (*N* = 75).

**Figure 3:**
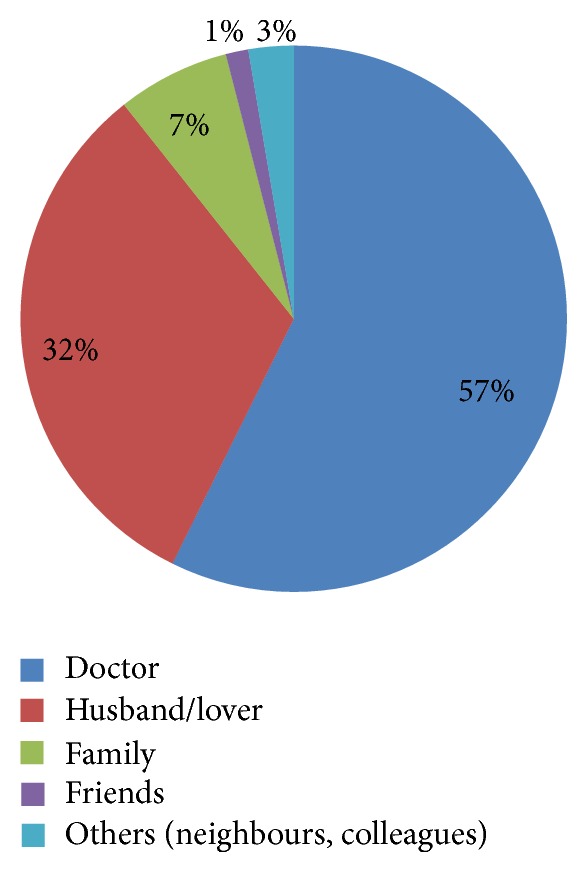
People who informed respondents' decisions to get treated (*N* = 75).

Figures 1, 2, and 3 show health seeking behaviour for antenatal depression among depressed respondents. With regard to the frequency distribution of who depressed respondents consulted when they were sad or lost interest in their routine activities, majority of the respondents chose husband/lover (68.9%), and this was followed by those who chose doctor (12.2%). With regard to the frequency distribution of the form of treatment they sought when they were sad or lost interest in routine activities for over one week, thirty-nine (52%) chose going for prayers in the church, and 31 (41.3%) chose hospital treatment. Forty-three (57.3%) and 24 (32%) mentioned doctor and husband/lover as those who informed their decisions to get treated, respectively.

## 4. Discussion

In this study, the prevalence of antenatal depression was 24.5%. This supports the finding of National Institute of Clinical Excellence which found prevalence of antenatal depression in developing countries to range from 19 to 25% [1]. A study in Malawi showed a prevalence rate of 21% [22] and another study in Ethiopia showed prevalence of 25% [23]. The reason for this high prevalence might be that most pregnant women with antenatal depression are not fully aware that they are depressed, and when some complain to their doctors about symptoms of antenatal depression, the doctors could mistake them for symptoms of other medical conditions as there is no screening for antenatal depression. It was observed in a study that antenatal depression is neither recognized nor adequately treated [9]. A cross-sectional study conducted in a rural South African area showed a high prevalence of antenatal depression, and it was 45% [24]. In Nigeria, a low prevalence, 9%, was observed in a study by Adewuya et al. [8].

In this study, the prevalence of antenatal depression in first, second, and third trimesters was 27.5%, 25.0%, and 23.5%, respectively. The prevalence of antenatal depression peaked in the first trimester and then gradually decreased across the second and third trimesters, albeit all trimesters having similar rates. Similar finding was observed by Gavin et al. who reported that prevalence of antenatal depression appears to peak in the first trimester [25].

The first-trimester prevalence of antenatal depression in this study corroborated the finding of a study with 357 pregnant women in Hong Kong in which antenatal depression prevalence was 22.1% [26] in the first trimester. This underscores the need to create awareness and educate pregnant women on the importance of registering early, especially in the first trimester, as this will provide them the opportunity to be screened for depression and those affected will be picked early for prompt treatment. The second trimester prevalence of antenatal depression in this study is equally high (25.0%). This is similar to a finding in Ethiopia with second-trimester prevalence of 27.6% [23], but lower than the prevalence of 43.2% among African American women reported in USA [27].

Furthermore, the prevalence of AD in the third trimester, though lower than it was in the first and second trimesters, is still high at 23.5%. This is supported by a similar finding observed in a cross-sectional study carried out with 292 Chinese people where prevalence of AD in the third trimester was 28.5% [28]. This is higher than the prevalence of AD in third trimester (8.3%) reported in a Nigerian study [8] and 37.8% among African American women in USA [27]. If depression is not treated at this stage, it might spill over to postpartum, resulting in postnatal depression.

Due to high prevalence of AD across the three trimesters, screening of pregnant women at each trimester should be established. This implies that a pregnant woman should be screened at least thrice for depression before childbirth, with one screening in each trimester. Education of risks associated with untreated antenatal depression should be initiated at various health facilities.

In this study, about ten risk factors were determined, out of which, three were identified as predictors of antenatal depression. The risk factors identified were attending antenatal care in public facilities, gender based abuse, drinking of alcohol in pregnancy, young age, presence of coexisting medical condition, history of previous caesarian section, unplanned pregnancy, single marital status, lack of education, and large family size.

In this study, attending a public health facility for antenatal care is a risk factor and predictor of antenatal depression. Though participants were not asked if the quality of service given in the public facilities affected them positively or negatively, some researchers in their studies identified possible reasons for public health facility being a risk factor. According to Mannava et al., who explored attitude and behaviour of health care workers towards their pregnant clients in Africa and Asia using secondary data from five electronic databases from January 1990 to December 2014, poor health care services were reported rendered in public health facilities. These poor services were long hours of waiting to see doctors or nurses due to high workload, absenteeism or unavailability of providers, verbal abuse, rudeness such as ignoring or ridiculing clients, neglect, authoritarian attitude, and frightening and unfriendly attitude of staff towards their pregnant clients [29]. Similar findings were observed in focal group discussion (FGD) involving women of child bearing age and men from two communities in Calabar and health staff of University of Calabar Teaching Hospital which found that negative staff attitudes towards patients stood as a barrier to the utilization of available obstetric care; and it was found that lack of incentives and inadequate materials and equipment to work with and poor remuneration contributed to negative staff attitudes [30]. These poor conditions can cause despair and stress to pregnant women and may result in depression. According to Karen Bruno, sustained stress leads to elevated hormones such as cortisol, “the stress hormone,” and reduced serotonin and other neurotransmitters in the brain. When the stress response fails to shut off and reset after a difficult situation has passed, it can lead to depression [31]. The difference in health care service between public and private health facilities in Nigeria was also observed in a study which reported that persistently low quality and inadequacy of health service provided in public facilities in Nigeria have made the private sector an unavoidable choice [32]. There should be disciplinary system in place to checkmate unfriendly and hostile staff especially in public health facilities. Provision of incentives to hospital staff, enhanced regular pay, and regular training of staff are suggested as possible solutions to hospital induced depression [30].

Young age (15–20 years) was identified as a risk factor of AD in this study. Young age as a risk factor was also identified in some studies [5, 33]. The reasons for this high prevalence of AD in young pregnant women might be that most are still single and financially dependent on their parents. In addition, some may lack support from their male partners. Some may be stigmatized by their parents, friends, and colleagues. All these could lead to depression and make some procure illegal abortions which might lead to infertility, perforated uterus, profuse bleeding per vagina, and death. Some might even think of committing suicide. Community education and awareness are very necessary to sensitize the populace on the need for their young pregnant daughters to register early for antenatal care. Effective social support should be established for this vulnerable group and family planning should be part of education to avoid occurrence or future recurrence of unintended pregnancy and large family which may bring about poverty.

Premarital pregnancy was also identified as a risk factor of antenatal depression in this study and this may also be related to the association between AD and young age. Prevalence of AD among pregnant single ladies was 50%. A study in USA observed a similar finding [5]. Reasons for this very high prevalence could be lack of support from family members and male partners, poor financial capacity, and having unwanted pregnancy. Education on increased risks of untreated depression should be intensified. Adequate social support system should be introduced.

Another risk factor identified in this study was unplanned pregnancy. In this study, prevalence of AD among pregnant women with unplanned pregnancy was 35.1%. Unplanned pregnancy has been reported in several studies as a risk factor of antenatal depression [5, 13, 34–36]. Reasons for the high prevalence of AD might be that most are not financially, psychologically, or socially prepared to cope with the demands of pregnancy; some are single and very young [37]; the married ones might be abused by their spouses; some were raped or forced to have sex; and some had contraceptive failure [38]. Education of risks of antenatal depression and family planning should be intensified.

Furthermore, lack of education was identified as a risk factor of AD in this study. Lack of education of woman as a risk factor was also identified in another study [35]. Illiteracy has been strongly linked to low self-esteem, feeling of worthlessness, and shame [39]. Government should introduce literacy education to these illiterate or poorly educated pregnant women to improve self-efficacy which has been demonstrated to lessen depression symptoms [39].

In this study, the presence of a medical condition in pregnancy was a risk factor for AD. Some of these conditions were chronic illnesses such as HIV, hypertension, and diabetes mellitus. This is similar to findings observed in some studies in KwaZulu Natal, South Africa, and Rio de Janeiro, Brazil [13, 40]. Depression is one of the complications of chronic illnesses. These illnesses cause tremendous life changes and limit mobility and independence. This can make pregnant women not do things they enjoy which can eat away self-confidence and sense of hope in the future. It comes as no surprise that they often feel despair and feel sad [41]. In this situation, early diagnosis and treatment of depression and coexisting medical conditions is very important. Social support should be encouraged by reaching out to family and friends.

Another important risk factor and predictor of antenatal depression in this study was gender based abuse. This is supported by a similar finding in a study in California, Los Angeles [42]. Prevalences of rape, physical assault, threats/intimidations, and poor financial support/financial deprivations (economic violence) experienced by respondents in this study were 1.9%, 5.7%, 5.1%, and 5.1%, respectively. The prevalence of rape in this study is supported by a similar finding observed in Abeokuta which reported 2.7% [43]. According to Fawole et al., prevalences of physical assault and threats to women were 10.8% and 6.8%, respectively [43]. The prevalence of financial abuse (economic violence) of women in this study was 5.1%. Data are very scarce to support or refute this finding. It is, therefore, no surprise to see connection between gender based abuse and depression as women who were and are still abused frequently experience depression.

Intake of alcohol in pregnancy was identified as a risk factor and predictor of antenatal depression in this study. Prevalence of alcohol intake in pregnancy was 5.8% in the study. This is well within a prevalence range of 1.9% and 11% observed in a study in Lagos, Nigeria [44]. Alcohol is a depressant. So, any amount of alcohol taken during pregnancy can lead to depression. No surprise that intake of alcohol in this study is a predictor of antenatal depression. Apart from depression, alcohol causes teratogenesis of the fetus, spontaneous abortion, preterm labour, low birth weight, and fetal alcoholic syndrome [44]. The study design in this study does not allow for temporality; thus it is difficult to know which comes first, depression or alcohol consumption. Alcohol is commonly used by those depressed to alleviate their mood. It is, therefore, necessary to embark on public health education on the effects of alcohol use in pregnancy through print and electronic media and community sensitization. There should be retraining of health workers on alcohol prevention counseling during pregnancy.

Going further, history of previous caesarian section was found to be a risk factor of AD. This is supported by a finding observed in a study in Navi Mumbai, India [36]. Women who have had a surgical birth are more likely to experience feelings of loss, personal failure, and low esteem [45]. Good supportive network that can address birth trauma issues is necessary. There is need to improve on facilities and manpower in secondary and tertiary health facilities as these will impact on the quality and safety of their services. Counseling and education of these vulnerable pregnant women on the importance of treatment of depression should be encouraged.

Another risk factor identified in this study is large family size (≥6 persons). This is similar to an inference made from another study on antenatal depression [5]. Depression is associated with large household with pronounced gender based violence cases [46]. Associated with large family size is low wealth index and social support. Social support and wealth index variables were not found to be significantly associated with antenatal depression in this study, but they showed important trends. Prevalence of antenatal depression was found to be high with low social support and low wealth index, and it decreased gradually with increasing social support and wealth index. Some studies reported significant associations between antenatal depression and low social support and low wealth index [5, 34, 35].

The health seeking behaviour for antenatal depression among women with AD was assessed and important inferences were drawn. Most participants reported consulting their husbands/lovers about symptoms of depression (68.9%), followed by those who reported consulting their doctors (12.2%), family members (10.8%), and friends (5.4%). This is supported by similar findings observed in Ohio, USA, in a study which reported that more depressed pregnant women consulted their family (especially their husbands and mothers) and friends about symptoms of depression than they consulted health care professionals [47]. Education about depression and treatment resources should be developed specifically to target social support persons especially the husband. These individuals are more likely to learn about women's symptoms than the health care provider because they are very close to these depressed women. On who influenced or informed their decisions to get treated, most reported being informed by their doctors to get treated (57.3%), followed by those who reported having their decisions to be treated being informed by their husbands (32%), family members (6.7%), and friends (1.7%). Similar inference was made in a study in Ohio, USA, but it was reported that mothers have greatest influence on their daughter's decisions to get treatment [47]. It is important to know that depressed women are likely to look for treatment if they share their illness symptoms with doctors, husbands, and, to a small extent, their family members. So, equipping doctors, husbands, and family members with knowledge about etiology, risk factors, and symptoms of depression through education will enable them to properly inform these women who need to get treated in the hospital.

On treatment preferences for depression, some reported going to church for prayers (52%) and some reported going to the hospital to get treated (41.3%). Only three (4%) reported self-medication. Contrary finding was reported in Ohio, USA, by Henshaw who reported that most depressed women sought treatment in hospitals, followed by, in a distant second position, self-treatment at home, and seeking help in place of worship came at a very distant third position [47]. The reason for these conflicting findings might be that many women in Abeokuta, nay Nigeria, are very religious. Indirect psychotherapy and social support obtained in these places of worship may be helping some women to cope with or reduce depressive symptoms. There is need to create awareness and educate clerics about depressive symptoms and dangers associated with untreated cases and to recognize worsening depressive conditions and steps for referral to hospitals for prompt treatment.

*Limitations of the Study*. The Edinburgh Postnatal Depression Scale (EPDS) is a screening tool and not a diagnostic tool. Any score equal to or above 12 is only suggestive of depression. So, to confirm any case of depression, referral to a psychiatric team should be done. Therefore, there should be a teamwork between obstetric team and psychiatric team.

Another limitation is that it was not possible to determine causal relationships between depression and risk factors but only to determine associations. This is due to the fact that the research was a cross-sectional study.

There may have been recall bias, underestimation, and overestimation of some experiences reported by the respondents but this was minimized by employing them to provide truthful responses and the fact that the information would be kept confidential and used for the purpose of the study alone.

## 5. Conclusions

In conclusion, findings in this study showed that prevalence of antenatal depression in Abeokuta North LGA is high at 24.5%. It peaked in the first trimester and slightly decreased with increasing trimester.

Various risk factors and predictors of antenatal depression were determined, and these were attending antenatal care in public facilities, gender based abuse or violence, intake of alcohol in pregnancy, young age, premarital pregnancy, unplanned pregnancy, illiteracy, history of previous caesarian section, coexisting medical conditions, and large family size.

Finally, the health seeking behaviour for antenatal depression among depressed women was determined. It was identified that involvement of clerics, husbands, family members, and friends in the management of antenatal depression was necessary to curb the menace. Some recommendations were made on how to tackle antenatal depression.

### 5.1. Implications for Public Health

The high prevalence of antenatal depression in this study is of public health concern; hence, health education and awareness campaigns should be embarked on to enlighten the populace about how to identify antenatal depression symptoms and the dangers of not getting it treated early. Edinburgh Postnatal Depression Scale (EPDS) screening should be introduced as part of antenatal care assessment in both private and public health facilities to help identify women with antenatal depression or at risk of developing it, and an invite should be sent to psychiatric team for comanagement. Measures such as education and awareness campaigns targeting reducing or prevention of risk factors should be embarked on. Successful reduction or elimination of risk factors will definitely reduce prevalence of antenatal depression.

### 5.2. Recommendations

Government and hospital management should introduce screening for depression as part of routine antenatal assessments in both public and private health facilities. Each pregnant woman should be screened at least thrice for depression before childbirth, with one screening in each trimester. Early registration for antenatal care especially in the first trimester should be encouraged since antenatal depression peaks in the first trimester. Community awareness campaigns should be embarked on by the state public health sector to educate the society on antenatal depression and its associated risk factors, dangers associated with untreated depression, and the need to get prompt help. Counseling on alcohol prevention should be initiated in all health facilities. Government should enact laws making gender based violence illegal and punishable as this would reduce prevalence of antenatal depression due to gender based violence.

Family planning should be encouraged by the doctors, especially after childbirth, to avoid unplanned and unwanted pregnancy and child spacing, limit family size, and prevent sexually transmitted diseases. Prompt treatment of coexisting medical conditions could help to reduce depressive symptoms and literacy education should be initiated by the state government as this has been found to reduce depressive symptoms. Social support network should be established which provides an avenue for all the at-risk and depressed women to come together and share their challenges and coping mechanisms. Further study is recommended, especially a community based one, to determine the gravity of antenatal depression in the community as this study might have underestimated the prevalence since it was hospital based.

## Figures and Tables

**Table 1:** Frequency distribution of the sociodemographic characteristics of the respondents.

Characteristics	Frequency (*n*)	Percentage (%)
*Age group *(*N* = 311)		
15–20 yrs (young)	34	10.9
21–35 yrs	255	82.0
36–49 yrs	22	7.1

*Ethnicity *(*N* = 314)		
Yoruba	308	98.1
Others	6	1.9

*Marital status *(*N* = 314)		
Single	18	5.7
Married	292	93.0
Others (separated or divorced)	4	1.3

*Level of education *(*N* = 311)		
No formal education	7	2.2
Primary	75	24.0
Secondary	158	50.6
Tertiary	72	23.1

*Type of family *(*N* = 293)		
Monogamous	230	78.5
Polygamous	61	20.8
Others	2	0.7

*Family size *(*N* = 296)		
Small (1–4 persons)	204	68.9
Average (5 persons)	41	13.9
Large (6 persons and above)	51	17.2

*Occupation *(*N* = 311)		
Civil servants	6	1.9
Unemployed	10	3.2
Students	13	4.2
Professional	34	10.9
Artisans	74	23.8
Traders	174	55.9

*Wealth index *(*N* = 314)		
Very poor	48	15.3
Poor	131	41.7
Average	70	22.3
Rich	55	17.5
Very rich	10	3.2

*Smoking cigarettes in this pregnancy? *(*N* = 298)		
Yes	1	0.3
No	297	99.7

*Drinking beer, wine, and/or spirit/liquor in this pregnancy? *(*N* = 294)		
Yes	17	5.8
No	277	94.2

*Kind of alcohol respondents take *(*N* = 17)		
Beer	5	29.4
Wine	6	35.3
Spirit/liquor	6	35.3

*Reasons for drinking alcohol in this pregnancy *(*N* = 17)		
For pleasure	15	88.2
Medicinal	2	11.8

*Social support (SSQN) *(*N* = 314)		
Low social support (SSQN < 1)	29	9.2
Average social support (SSQN = 1)	216	68.8
High social support (SSQN > 1)	69	22.0

*Gender based abuse *(*N* = 314)		
Rape	6	1.9
Intimidation/threat	16	5.1
Inadequate financial support/financial deprivation	16	5.1
Physical assault	18	5.7
None	258	82.2

*Childhood experience *(*N* = 314)		
Pleasant (enjoyable)	239	76.1
Unpleasant (gloomy)	17	5.4
I do not know	58	18.5

**Table 2:** Frequency distribution of respondents by history of index pregnancy and past gynaecological/obstetric history among respondents.

Variables	Frequency (*n*)	Percentage (%)
*Age of pregnancy in months *(*N* = 313)		
First trimester (1–3 months)	51	16.3
Second trimester (4–6 months)	92	29.4
Third trimester (7–9 months)	170	54.3

*Was the pregnancy expected (planned)? *(*N* = 313)		
Yes	236	75.4
No	77	24.6

*Coexisting medical conditions *(*N* = 305)		
Hypertension	2	0.7
Diabetes	1	0.3
HIV	5	1.6
Others^*∗*^	4	1.3
No coexisting condition	293	96.1

*Gravidity *(*N* = 309)		
Primigravida	71	23.0
Multigravida	238	77.0

*Parity *(*N* = 307)		
Nullipara	91	29.6
Primipara	93	30.3
Multipara	123	40.1

*Abortions/miscarriages *(*N* = 303)		
None	218	71.9
One or more	85	28.1

*Sex of baby*		
Male	172	47.9
Female	187	52.1

*Mode of deliveries*		
Caesarian section	10	2.8
Vaginal delivery	349	97.2

*Babies' weight*		
Low birth weight (<2.5 kg)	6	1.7
Normal weight (2.5–4.0 kg)	351	97.8
High birth weight (>4.0 kg)	2	0.6

*Breastfed?*		
Yes	359	100.0
No	0	0.0

^*∗*^Sickle cell, epilepsy, and UTI.

**Table 3:** Associations between sociodemographic variables and antenatal depression.

Characteristics	Depressed *n* (%)	Nondepressed *n* (%)	Total (%)	*χ* ^2^	*P* value
*Level of care* (*N* = 314)^*∗*^					
Primary	28 (26.4)	78 (73.6)	106 (100)		
Secondary	13 (12.5)	91 (87.5)	104 (100)	14.051	0.001
Tertiary	36 (34.6)	68 (65.4)	104 (100)		

*Type of health facility* ^*∗*^ (*N* = 314)					
Public health facilities	55 (35.3)	101 (64.7)	156 (100)	19.300	0.000
Private health facilities	22 (13.9)	136 (86.1)	158 (100)		

*Age group* (*N* = 311)^*∗*^					
15–20 years (young)	15 (44.1)	19 (55.9)	34 (100)		
21–35 years	58 (22.7)	197 (77.3)	255 (100)	8.917	0.012
36–49 years (elderly)	3 (13.6)	19 (86.4)	22 (100)		

*Marital status* (*N* = 314)^*∗*^					
Single	9 (50)	9 (50)	18 (100)	6.697	0.010
Married/others^*∗∗*^	68 (23)	228 (77)	296 (100)		

*Level of education* (*N* = 311)^*∗*^					
No formal education	5 (71.4)	2 (28.6)	7 (100)		
Primary	17 (22.7)	58 (77.3)	75 (100)	9.592	0.022
Secondary	41 (25.9)	117 (74.1)	158 (100)		
Tertiary	14 (19.4)	58 (80.6)	72 (100)		

*Occupation* (*N* = 311)					
Professionals	6 (17.6)	28 (82.4)	34 (100)		
Civil servants	1 (16.7)	5 (83.3)	6 (100)		
Artisans	17 (23)	57 (77)	74 (100)	3.837	0.429
Traders	44 (25.3)	130 (74.7)	174 (100)		
Students/unemployed	9 (39.1)	14 (60.9)	23 (100)		

*Type of family* (*N* = 293)					
Monogamy	53 (23)	177 (77)	230 (100)	0.019	0.891
Polygamy/others	14 (22.2)	49 (77.8)	63 (100)		

*Family size* (*N* = 296)^*∗*^					
Small family size (1–4 persons)	49 (24)	155 (76)	204 (100)		
Average family size (5 persons)	4 (9.8)	37 (90.2)	41 (100)	7.047	0.029
Large family size (≥6 persons)	17 (33.3)	34 (66.7)	51 (100)		

*Wealth index* (*N* = 314)					
Very poor	14 (29.2)	34 (70.8)	48 (100)		
Poor	34 (26)	97 (74)	131 (100)		
Average	16 (22.9)	54 (77.1)	70 (100)	1.528	0.822
Rich	11 (20)	44 (80)	55 (100)		
Very rich	2 (20)	8 (80)	10 (100)		

*Drinking beer, wine, and/or spirit/liquor in this pregnancy?* (*N* = 294)^*∗*^					
Yes	9 (52.9)	8 (47.1)	17 (100)	8.441	0.004
No	61 (22)	216 (780)	277 (100)		

*Social support (SSQ)* (*N* = 314)					
Low social support	10 (34.5)	19 (65.5)	29 (100)		
Average social support	53 (24.5)	163 (75.5)	216 (100)	2.222	0.329
High social support	14 (20.3)	55 (79.7)	69 (100)		

*Gender based abuse* (*N* = 314)^*∗*^					
Rape	3 (50)	3 (50)	6 (100)		
Physical assault	9 (50)	9 (50)	18 (100)		
Intimidation/threat	8 (50)	8 (50)	16 (100)	18.636	0.001
Financial deprivation/poor	6 (37.5)	10 (62.5)	16 (100)		
Financial support					
None	51 (19.8)	207 (80.2)	258 (100)		

*Childhood experience* (*N* = 256)					
Pleasant	58 (24.3)	181 (75.7)	239 (100)	2.395	0.122
Unpleasant	7 (41.2)	10 (58.8)	17 (100)		

^*∗*^Significant variables.

^*∗∗*^Divorced and separated.

**Table 4:** Associations between obstetric/gynaecological variables and antenatal depression.

Characteristics	Depressed *n* (%)	Nondepressed *n* (%)	Total (%)	*χ* ^2^	*P* value
*Age of pregnancy in months *(*N* = 313)					
First trimester (1–3 months)	14 (27.5)	37 (72.5)	51 (100)		
Second trimester (4–6 months)	23 (25)	69 (75)	92 (100)	0.336	0.845
Third trimester (7–9 months)	40 (23.5)	170 (76.5)	170 (100)		

*Was the pregnancy expected (planned)?* (*N* = 313)^*∗*^					
Yes	50 (21.2)	186 (78.8)	236 (100)	6.029	0.014
No	27 (35.1)	50 (64.9)	77 (100)		

*Coexisting medical conditions* (*N* = 305)^*∗*^					
Present	6 (50)	6 (50)	12 (100)	4.503	0.034
Absent	68 (23.2)	225 (76.8)	293 (100)		

*Gravidity* (*N* = 309)					
Primigravida	18 (25.5)	53 (74.5)	71 (100)	0.059	0.809
Multigravida	57 (23.9)	181 (76.1)	238 (100)		

*Parity* (*N* = 307)					
Nullipara	18 (19.8)	73 (80.2)	91 (100)		
Primipara	27 (29)	66 (71)	93 (100)	2.136	0.344
Multipara	31 (24.2)	92 (75.8)	123 (100)		

*Abortions/miscarriages* (*N* = 303)					
None	56 (25.7)	162 (74.3)	218 (100)	0.365	0.546
One or more	19 (22.4)	66 (77.6)	85 (100)		

*History of previous caesarian sections* (*N* = 194)^*∗*^					
Yes	5 (55.6)	4 (44.4)	9 (100)	4.589	0.032
No	44 (23.8)	141 (76.2)	185 (100)		

^*∗*^Significant.

**Table 5:** Results of multiple regression for predictors of antenatal depression.

Variables	Unadjusted OR	95% CI	Adjusted OR	95% CI
*Age*				
15–20 years (ref)	1.00			
21–35 years	2.68	1.28–5.61		
36–49 years	5.00	1.24–20.14		

*Level of education*				
No formal education (ref)	1.00			
Primary	8.53	1.52–47.95		
Secondary	7.13	1.33–38.20		
Tertiary	10.36	1.82–59.04		

*Marital status*				
Single (ref)	1.00			
Married/others^*∗∗*^	3.42	1.31–8.98		

*Family size*				
Small family size (1–4 persons)	1.58	0.81–3.08		
Average family size (5 persons)	4.63	1.42–15.12		
Large family size (≥6 persons) (ref)	1.00			

*Type of facility* ^*∗*^				
Public health facilities (ref)	1.00			
Private health facilities	3.37	1.93–5.88	5.00	2.52–9.89

*Intake of alcohol* ^*∗*^				
Yes (ref)	1.00			
No	3.98	1.48–10.76	5.05	1.71–14.94

*Gender based abuse* ^*∗*^				
Present (ref)	1.00			
Absent	3.90	2.10–7.24	4.31	2.09–8.88

*Was pregnancy expected?*				
Yes	2.01	1.14–3.53		
No (ref)	1.00			

*Coexisting medical conditions*				
Present (ref)	1.00			
Absent	3.31	1.03–10.59		

*History of previous caesarian sections *				
Yes (ref)	1.00			
No	4.01	1.03–15.57		

^*∗*^Predictors.

^*∗∗*^Divorced and separated.
